# Supplementary material for: APOE ε4 in Depression-Associated Memory Impairment—Evidence from Genetic and MicroRNA Analyses
Source: Biomedicines. 2022 Jun 30;10(7):1560. doi: 10.3390/biomedicines10071560 (PMC9313258; doi:10.3390/biomedicines10071560)
Supplement: Supplementary file 1 [file biomedicines-10-01560-s001.zip › biomedicines-1760839-supplementary.pdf]

SUPPLEMENT

APOE ε4 in Depression-Associated Memory Impairment— Evidence from Genetic and MicroRNA Analyses

Sarah Bonk, Kevin Kirchner, Sabine Ameling , Linda Garvert, Henry Völzke, Matthias Nauck, Uwe Völker, Hans J. Grabe and Sandra Van der Auwera

Preparation and profiling of plasma miRNAs

Non-cellular blood circulating miRNAs were prepared from 200 µl EDTA plasma using the miRCURY™ RNA Isolation Kit –Biofluids (Qiagen, Hilden, Germany). As described in Ameling et al, 2015, the laboratory workflow involved several quality control steps. First, to monitor RNA isolation and ensure high and reproducible RNA yield from EDTA-plasma samples, RNA-spike-Ins (UniSp2, UniSp4, and UniSp5) and bacteriophage MS2 carrier RNA were added to each sample at the beginning of the purification procedure. Reverse transcription reactions were performed using Universal cDNA Synthesis Kit II (Qiagen, Hilden, Germany) with addition of Spike-Ins (Cel-miR-39 and UniSp6) during cDNA synthesis according to the manufacturer's instructions. Before using RNA samples for miRNA profiling, the presence of Spike-Ins, yield of typical plasma miRNAs, absence of PCR inhibitors as well as hemolysis in the samples was assessed by use of a microRNA QC PCR Panel V1.M (Qiagen, Hilden, Germany). For RT-qPCR based miRNA analysis the pre-defined Serum/ Plasma Focus microRNA PCR Panels V3.M and V4.M (Qiagen, Hilden, Germany) were used, covering 179 miRNAs detectable in biofluids (miRBase version 20). The qPCR of the first batch of SHIP-TREND miRNA samples was performed on the 7900HT Real-time PCR system and the second batch was performed on the QuantStudio™ 12K Flex Real-Time PCR system (both Thermo Fisher scientific, Waltham, USA). To determine the technical variation between the Serum/Plasma Focus microRNA PCR Panel plates, the interpolate calibrator (IPC) (UniSp3) was analysed (Batch 1: 20.9 ± 0.2, Batch 2: 18.6 ± 0.3 Ct values

of the IPC were 20 ± 0.2 (mean ± SD) across all samples, and thus highly similar. Cycling parameters as recommended by Qiagen were employed in 42 amplification cycles (Polymerase Activation/Denaturation 95˚C, 10 min, Amplification at 95˚C, 10 s, 60˚C, 1 min, ramp-rate 1.6˚C/s). The amplification curves were analyzed using the Quantstudio 12 K Flex software Real-time PCR software and SDS 2.4 software (both Thermo Fisher Scientific, Waltham, USA) for determination of Ct (threshold cycle: that

PCR cycle where the target is quantified according to Real-time PCR data Markup Language (RDML) guidelines) and for melting curve analysis.

Micro RNAs available for analysis

hsa-let-7b_3p, *hsa*-let-7b-5p, *hsa* -let-7c-5p, *hsa* -let-7d-3p, *hsa as*-let-7d-5p, *hsa* -let-7e-5p, *hsa* -let-7f-5p, *hsa* -let-7g-5p, *hsa* -let-7i-5p, *hsa* -miR-101-3p, *hsa* -miR-103a-3p, *hsa* -miR-106a-5p, *hsa as*-miR-106b-3p, *hsa* -miR-106b-5p, *hsa* -miR-107, *hsa as*-miR-10b-5p, *hsa* -miR-122-5p, *hsa* -miR-125a-5p, *hsa* -miR-125b-5p, *hsa* -miR-126-3p, *hsa* -miR-130a-3p, *hsa* -miR-130b-3p, *hsa* -miR-132-3p, *hsa* -miR-133a-3p, *hsa*-miR-133b, *hsa* -miR-136-5p, *hsa* -miR-139-5p, *hsa as*-miR-140-3p, *hsa* -miR-140-5p, *hsa* -miR-142-3p, *hsa* -miR-142-5p, *hsa* -miR-143-3p, *hsa* -miR-144-3p, *hsa* -miR-144-5p, *hsa* -miR-145-5p, *hsa* -miR-146a-5p, *hsa* -miR-146b-5p, *hsa* -miR-148a-3p, *hsa* -miR-148b-3p, *hsa* -miR-150-5p, *hsa* -miR-151a-3p, *hsa* -miR-151a-5p, *hsa* -miR-152-3p, *hsa* -miR-154-5p, *hsa* -miR-155-5p, *hsa* -miR-15a-5p, *hsa* -miR-15b-3p, *hsa* -miR-15b-5p, *hsa* -miR-16-2-3p, *hsa* -miR-16-5p, *hsa* -miR-17-5p, *hsa* -miR-181a-5p, *hsa* -miR-185-5p, *hsa* -miR-186-5p, *hsa* -miR-18a-5p, *hsa* -miR-18b-5p, *hsa* -miR-191-5p, *hsa* -miR-192-5p, *hsa* -miR-194-5p, *hsa* -miR-195-5p, *hsa* -miR-197-3p, *hsa* -miR-199a-3p, *hsa* -miR-199a-5p, *hsa* -miR-19a-3p, *hsa* -miR-19b-3p, *hsa* -miR-205-5p, *hsa* -miR-20a-5p, *hsa* -miR-21-5p, *hsa* -miR-210-3p, *hsa* -miR-2110, *hsa* -miR-215-5p, *hsa* -miR-22-3p, *hsa* -miR-22-5p, *hsa* -miR-221-3p, *hsa* -miR-222-3p, *hsa* -miR-223-3p, *hsa* -miR-223-5p, *hsa* -miR-23a-3p, *hsa* -miR-23b-3p, *hsa* -miR-24-3p, *hsa* -miR-25-3p, *hsa* -miR-26a-5p, *hsa* -miR-26b-5p, *hsa* -miR-27a-3p, *hsa* -miR-27b-3p, *hsa* -miR-28-3p, *hsa* -miR-28-5p, *hsa as*-miR-29a-3p, *hsa* -miR-29b-3p, *hsa* -miR-29c-3p, *hsa*-miR-301a-3p, *hsa* -miR-30a-5p, *hsa* -miR-30b-5p, *hsa* -miR-30c-5p, *hsa* -miR-30d-5p, *hsa* -miR-30e-3p, *hsa* -miR-30e-5p, *hsa* -miR-32-5p, *hsa* -miR-320a, *hsa*-miR-320b, *hsa*-miR-324-3p, *hsa* -miR-324-5p, *hsa* -miR-326, *hsa* -miR-328-3p, *hsa* -miR-331-3p, *hsa* -miR-335-5p, *hsa* -miR-338-3p, *hsa* -miR-339-3p, *hsa* -miR-339-5p, *hsa* -miR-33a-5p, *hsa* -miR-342-3p, *hsa* -miR-34a-5p, *hsa* -miR-363-3p, *hsa* -miR-365a-3p, *hsa* -miR-374a-5p, *hsa* -miR-374b-5p, *hsa* -miR-375, *hsa* -miR-376a-3p, *hsa* -miR-382-5p, *hsa* -miR-409-3p, *hsa* -miR-421, *hsa* -miR-423-3p, *hsa* -miR-423-5p, *hsa* -miR-424-5p, *hsa* -miR-425-3p, *hsa* -miR-425-5p, *hsa* -miR-451a, *hsa* -miR-484, *hsa* -miR-485-3p, *hsa* -miR-486-5p, *hsa* -miR-495-3p, *hsa* -miR-497-5p, *hsa* -miR-501-3p, *hsa* -miR-502-3p, *hsa* -miR-505-3p, *hsa* -miR-532-3p, *hsa* -miR-532-5p, *hsa* -miR-543, *hsa* -miR-551b-3p, *hsa* -miR-574-3p, *hsa* -miR-584-5p, *hsa* -miR-590-5p, *hsa* -miR-605, *hsa* -miR-629-5p, *hsa* -miR-652-3p, *hsa* -miR-660-5p, *hsa* -miR-766-3p, *hsa* -miR-885-5p, *hsa* -miR-92a-3p, *hsa* -miR-93-3p, *hsa* -miR-93-5p, *hsa* -miR-99a-5p, *hsa* -miR-99b-5p, *hsa* -miR-100-5p, *hsa* -miR-126-5p, *hsa* -miR-1260a, *hsa* -miR-128-3p, *hsa* -miR-136-3p, *hsa* -miR-193a-5p, *hsa* -miR-320c, *hsa* -miR-320d, *hsa* -miR-335-

3p, *hsa* -miR-361-5p, *h hsa as*-miR-362-3p, *hsa* -miR-376c-3p, *hsa*-miR-454-3p, *hsa*-miR-483-5p, *hsa* -miR-7-1-3p, *hsa* -miR-874-3p, *hsa*-miR-877-5p

Table S1. Sample characteristic and comparison of the two miRNA batches. For metric variables mean, sd and range are given, for categorical variables numbers and percentage are given.

Variable	Batch 1 (n=371)	Batch 2 (n=337)	Comparison
**Age**	49.7 (13.8), [22-79]	50.3 (13.7), [21-79]	T=-0.57, p=0.57
**Sex**			Chi2=0.008, p=0.93
**Males**	187 (50.4%)	171 (50.7%)	
**Females**	184 (49.6%)	166 (49.3)	
**Education**			Chi2=0.64, p=0.73
**<10 years**	41 (11.0%)	31 (9.3%)	
**=10 years**	211 (56.9%)	193 (57.6%)	
**>10 years**	119 (32.1%)	111 (33.1%)	
**Smoking status**			Chi2=0.51, p=0.77
**never**	151 (40.7%)	142 (42.3%)	
**ever**	138 (37.2%)	127 (37.8%)	
**current**	82 (22.1%)	67 (19.9%)	
**BMI**	27.5 (4.4), [17.7-48.1]	27.2 (4.2), [18.5-42.3]	T=1.04, p=0.30
**Depressive symptoms (PHQ-9)**	12.7 (3.2), [9-31]	12.8 (3.8), [9-35]	-0.64, p=0.52
**Mental health score (MCS)**	53.5 (7.2), [22-66]	52.6 (8.7), [17-66]	T=1.38, p=0.17
**Hematocrit (HCT)**	0.42 (0.03), [0.28-0.52]	0.42 (0.03), [0.34-0.51]	T=-2.16, p=0.03
**Platelet count (PLT)**	226.9 (49.0), [102-390]	226.7 (49.3), [93-392]	T=0.04, p=0.96
**APOE ε4 status**			Chi2=2.82, p=0.24
**ε4 carrier**	87 (24.5%)	77 (23.6%)	

mean, standard deviation and range for metric variables; numbers and percentage for categorical variables; PHQ: Patients Health Questionnaire, MCS: Mental Health Composite Score.
